# Matrix metalloproteinase-9 (MMP9) is involved in the TNF-α-induced fusion of human M13SV1-Cre breast epithelial cells and human MDA-MB-435-pFDR1 cancer cells

**DOI:** 10.1186/s12964-018-0226-1

**Published:** 2018-04-10

**Authors:** Julian Weiler, Marieke Mohr, Kurt S. Zänker, Thomas Dittmar

**Affiliations:** 1 Institute of Immunology, Centre of Biomedical Education and Research (ZBAF), Witten/Herdecke University, Stockumer Str. 10, 58448 Witten, Germany; 2 BioGenes GmbH, Köpenicker Str. 325, 12555 Berlin, Germany

**Keywords:** Cell fusion, TNF-α, Minocycline, MMP9, Breast cancer

## Abstract

**Background:**

In addition to physiological events such as fertilisation, placentation, osteoclastogenesis, or tissue regeneration/wound healing, cell fusion is involved in pathophysiological conditions such as cancer. Cell fusion, which applies to both the proteins and conditions that induce the merging of two or more cells, is not a fully understood process. Inflammation/pro-inflammatory cytokines might be a positive trigger for cell fusion. Using a *Cre-LoxP*-based cell fusion assay we demonstrated that the fusion between human M13SV1-Cre breast epithelial cells and human MDA-MB-435-pFDR1 cancer cells was induced by the pro-inflammatory cytokine tumour necrosis factor-α (TNF-α).

**Methods:**

The gene expression profile of the cells in the presence of TNF-α and under normoxic and hypoxic conditions was analysed by cDNA microarray analysis. cDNA microarray data were verified by qPCR, PCR, Western blot and zymography. Quantification of cell fusion events was determined by flow cytometry. Proteins of interest were either blocked or knocked-down using a specific inhibitor, siRNA or a blocking antibody.

**Results:**

The data showed an up-regulation of various genes, including claudin-1 (CLDN1), ICAM1, CCL2 and MMP9 in M13SV1-Cre and/or MDA-MB-435-pFDR1 cells. Inhibition of these proteins using a blocking ICAM1 antibody, CLDN1 siRNA or an MMP9 inhibitor showed that only the blockage of MMP9 was correlated with a decreased fusion rate of the cells. Likewise, the tetracycline-based antibiotic minocycline, which exhibits anti-inflammatory properties, was also effective in both inhibiting the TNF-α-induced MMP9 expression in M13SV1-Cre cells and blocking the TNF-α-induced fusion frequency of human M13SV1-Cre breast epithelial cells and human MDA-MB-435-pFDR1 cancer cells.

**Conclusions:**

The matrix metalloproteinase-9 (MMP9) is most likely involved in the TNF-α-mediated fusion of human M13SV1-Cre breast epithelial cells and human MDA-MB-435-pFDR1 cancer cells. Likewise, our data indicate that the tetracycline-based antibiotic minocycline might exhibit anti-fusogenic properties because it inhibits a cell fusion-related mechanism.

**Electronic supplementary material:**

The online version of this article (10.1186/s12964-018-0226-1) contains supplementary material, which is available to authorized users.

## Background

Even though cell fusion has a pivotal role in several physiological and pathophysiological conditions such as fertilisation, placentation, muscle development, osteoclastogenesis, wound healing, tissue regeneration, infection with enveloped viruses, and cancer (for review see: [1–4]), the conditions that favour and the detailed mechanisms of how the plasma membranes of two or more cells merge are not fully understood.

During evolution different cell fusion strategies developed using different proteins and protein-protein interactions. Some cell fusion-mediating proteins, such as EFF-1 in *C. elegans* and members of the syncytin family (syncytin-1 and syncytin-2 in humans or *syncytin-A* and *syncytin–B* in mice) are of viral and retroviral origin (for review see: [1, 5, 6]). Because of the high homology of these proteins to viral fusion proteins, the cell fusion mechanisms might conceivably be similar. Syncytin-1 and syncytin-2 are expressed in trophoblasts and mediate their fusion to multi-nucleated syncytiotrophoblasts [5]. Trophoblast fusion depends on cyclic AMP (cAMP)/PKA- and PKC-dependent mechanisms [7]. In contrast, the fusion of myoblasts to multinucleated myofibres chiefly depends on the reorganisation of the actin cytoskeleton and the interplay of several actin regulatory proteins (for review see: [8]). In order to fuse with osteoclasts, the macrophages first have to adopt a fusion-competent status, which is induced through several cytokines, including IL-4, IL-13, M-CSF and RANKL, and surface molecules such as TREM-2 and DAP12 (for review see: [9]). Fusion-competent macrophages are characterised by the expression of several markers, including E-cadherin, DC-STAMP/OC-STAMP, CD200, SIRPα, CD9 and CD81, CCL2, and MMP9 (for review see: [9]). The fusion of mesenchymal stem cells with MDA-MB-231 breast cancer cells depends on S100A4 [10].

The induction of a fusion-competent status indicates that the cells per se are not fusogenic, but rather, have to acquire the ability to fuse, which has been termed priming [4]. In addition to priming, four additional steps have been proposed for the entire cell fusion process, namely, chemotaxis, adhesion, fusion and post-fusion (an excellent and detailed overview is given in [4]). Fusion-competent cells have to migrate towards their cellular fusion partners by chemotaxis and close cell-cell contact is a prerequisite for the subsequent fusion step. As important as the induction of a fusion-competent status is, fused cells must finally acquire a fusion-incompetent status to prevent further cell fusion events.

Even though factors have been identified that induce a fusion-competent status in macrophages, the regulation of the entire process but particularly basal cell fusion events that occur between different body cell types, is not understood. In addition to macrophages [11], basal cell fusion events were also observed for bone marrow-derived stem cells (BMDCs) [12], intestinal cells [13], and haematopoietic cells [14]. It is unclear how these spontaneous cell fusion events are regulated. However, the data from several studies indicated an increased cell fusion frequency under injurious/inflammatory conditions. For instance, the fusion frequency of bone marrow-derived stem cells (BMDCs) with epithelial intestinal cells and Purkinje neurons is markedly increased in response to chronic inflammation [13, 15]. Likewise, an increased fusion frequency for different cell types, including macrophages and tumour cells, was observed in the presence of the pro-inflammatory cytokine TNF-α [16–19]. In this context, it is worth speculating whether the chronically inflamed tumour microenvironment [20, 21] would also provide a fusion-friendly milieu. It is well-known that tumour cells could fuse with tumour cells and normal cells, like macrophages and stem cells, thereby giving rise to hybrid cells that could exhibit novel properties, such as an enhanced metastatic capacity and an increased drug resistance (for review see: [22–24]).

To clarify whether the fusion of tumour cells and normal cells is triggered by cytokines, chemokines and/or growth factors, we developed a *Cre-LoxP*-based cell fusion assay [18]. Human breast cancer cells were stably transfected with a fluorescence double reporter (FDR) plasmid containing a *loxP* flanked HcRed/DsRed cassette followed by an EGFP cassette and were co-cultured with *Cre* recombinase-expressing human breast epithelial cells [18]. The Cre-mediated recombination in fused cells led to a switch from red fluorescence to green fluorescence that could be easily quantified by flow cytometry [18]. Using this assay we were able to show that the fusion of human breast cancer cells and human breast epithelial cells was positively triggered by TNF-α in a dose-dependent manner [18].

Here, we investigated the impact of TNF-α-induced protein expression on cell fusion. MMP9 was markedly up-regulated in TNF-α treated cells and the inhibition of MMP9 activity was correlated with a significantly impaired TNF-α-induced fusion rate. Similar results were achieved using the tetracycline-based antibiotic minocycline, which also has anti-inflammatory properties (for review see: [25]).

## Methods

### Cell culture

M13SV1-Cre-Puro human breast epithelial cells and MDA-MB-435-pFDR1 human cancer cells were generated as previously described [18]. The pFDR1 vector [26] was kindly provided by Frank Edenhofer (University of Würzburg, Würzburg, Germany). M13SV1-mCherry-Cre human breast epithelial cells were derived from M13SV1 human breast epithelial cells [27] by stable transduction with the pcDNA-mCherry-P2A-Cre vector. This vector was derived by excising the mCherry-P2A-Cre sequence of pLM-CMV-R-Cre (a gift from Michel Sadelain (Addgene plasmid #27546)) with NheI and SalI (both restriction enzymes were purchased from ThermoFisher Scientific GmbH, Schwerte, Germany) and cloning it into the pcDNA3.1 vector digested with NheI and XhoI (vector and restrictions enzymes were obtained from ThermoFisher Scientific GmbH, Schwerte, Germany). XhoI and SalI have identical cohesive ends. M13SV1-Cre-Puro and M13SV1-mCherry-Cre human breast epithelial cells (M13SV1-Cre cells) were cultivated in MSU-1 basal media (Biochrom GmbH, Berlin, Germany) supplemented with 10% foetal calf serum (FCS; Biochrom GmbH, Berlin, Germany), 1% penicillin/streptomycin (100 U/ml penicillin, 0.1 mg/ml streptomycin; Sigma-Aldrich, Taufkirchen, Germany), 10 μg/ml human recombinant EGF, 5 μg/ml human recombinant insulin, 0.5 μg/ml hydrocortisone, 4 μg/ml human transferrin, 10 nM β-oestrogen (all chemicals were purchased from Sigma-Aldrich, Taufkirchen, Germany) and 1 μg/ml puromycin (InvivoGen, Toulouse, France). MDA-MB-435-pFDR1 cells were cultivated in DMEM media (Sigma-Aldrich, Taufkirchen, Germany) supplemented with 10% FCS (Biochrom GmbH, Berlin, Germany), 1% penicillin/ streptomycin (100 U/ml penicillin, 0.1 mg/ml streptomycin; Sigma-Aldrich, Taufkirchen, Germany), and 2 μg/ml puromycin (InvivoGen, Toulouse, France). All cells were maintained in a humidified atmosphere at 37 °C and 5% CO_2_.

### Cell fusion assay

Quantification of fusion events between M13SV1-Cre cells and MDA-MB-435-pFDR1 cells was performed as previously described [18]. Briefly, M13SV1-Cre human breast epithelial cells and MDA-MB-435-pFDR1 human breast cancer cells were co-cultured in a ratio of 1:3 per well of a 96-well plate for 72 h in a humidified atmosphere at 37 °C and 5% CO_2_. Depending on the experiment 100 ng/ml TNF-α (Bio-Techne GmbH, Wiesbaden-Nordenstadt, Germany), up to 10 μg/ml minocycline (Sigma-Aldrich, Taufkirchen, Germany), up to 10 μM SB-3CT (Sigma Aldrich, Taufkirchen, Germany), 10 μg/ml anti-ICAM1/CD54 (mouse monoclonal, clone 11C81; Bio-Techne GmbH, Wiesbaden-Nordenstadt, Germany) and appropriate combinations of inhibitors and TNF-α were added to the cell culture media. Hypoxia was induced by culturing the cells in stepwise oxygen-deprivation conditions (24 h with 10% O_2_, 24 h with 5% O_2_, 24 h with 1% O_2_) in hypoxia chambers (Billups-Rothenberg, Del Mar, CA, USA) with a constant flow of 5% CO_2_ and the remainder of nitrogen, as previously described [18]. All co-cultured cells were harvested, washed once with PBS and the amount of EGFP-expressing cells was quantified by flow cytometry (FACSCalibur; Becton Dickenson, Heidelberg, Germany). Freshly harvested M13SV1-Cre cells and MDA-MB-435-pFDR1 cells mixed in a ratio of 1:3 served as a negative control to adjust the flow cytometer. The relative fold change was calculated in relation to untreated co-cultured cells, which was set to 1. Each condition was assayed in triplicate.

### Total RNA preparation

Total RNA from all the cell lines/cells used in this study was isolated using the NucleoSpin® RNA Kit (Macherey Nagel, Düren, Germany) according to the manufacturer’s instructions. For PCR and qPCR, RNA concentration and purity was determined by UV spectrophotometric analysis. For subsequent cDNA microarray studies, the RNA integrity number (RIN) was determined using an Agilent 2100 Bioanalyzer (Agilent Technologies, Waldbronn, Germany). The samples were diluted to a final concentration of 500 ng/μl and applied to an RNA chip according to the manufacturer’s instructions. RNA samples with a RIN of 8 to 10 were used for microarray analysis.

### Single-colour microarray analysis

To investigate differential gene expression after the TNF-α stimulation of MDA-MB-435-pFDR1 and M13SV1-Cre-Puro cells under normoxia and hypoxia, a single colour microarray analysis (Agilent Human 4×44K v2 Microarray (Agilent Technologies, Waldbronn, Germany)) was performed. The total RNA of the cells (three independent experiments were pooled) matching the RIN criteria of 8 to 10 were sent on dry ice to Source BioSciences (Nottingham, UK), which performed synthesis and Cy3 labelling of the cDNA and hybridisation of the microarrays. Microarray gene expression data were analysed using GeneSpring GX v14.8 software (Agilent Technologies, Waldbronn, Germany). Expression data were normalized based on quantiles with a threshold of 1 and no baseline transformation was performed. For further analysis, data were filtered by flags (not detected flags and compromised spots were removed) and fold changes (2-fold up and down). All microarray data were deposited in the ArrayExpress database at EMBL-EBI (https://www.ebi.ac.uk/arrayexpress) under accession number E-MTAB-6084.

### RT-PCR and qPCR

Synthesis of cDNA from mRNA by reverse transcription was performed using the RevertAid First Strand cDNA Synthesis Kit (ThermoFisher Scientific GmbH, Schwerte, Germany) as referred to in the instruction manual. Conventional PCR (total reaction volume of 25 μl) was performed with ready to use 5× Master Mix containing Bio&Sell Taq-Polymerase, dNTPs and MgCl_2_ (Bio&Sell GmbH Nuremberg, Germany) and 10 μM primers (ThermoFisher Scientific GmbH, Schwerte, Germany). Cycling conditions comprised of an initial denaturation for 5 min at 94 °C and 30 cycles of 30 s at 94 °C, 30 s at the appropriate annealing temperature and 30 s at 72 °C followed by final elongation for 7 min at 72 °C. Primer pairs used in this study are summarised in Table 1. PCR products were separated on a 1.5% agarose gel and the bands were visualized with GelRed™ stain (VWR International GmbH, Darmstadt, Germany) and the GFelDoc™ EZ Imager system (Bio-Rad, Munich, Germany). For qPCR (total volume of 10 μl per reaction), the SYBR Green Super Mix with ROX (Quanta Bioscience, Beverly, CA, USA) and 10 μM primers (Table 1) were used according to the manufacturer’s instructions. The StepOne Plus Real-Time PCR System (ThermoFisher Scientific GmbH, Schwerte, Germany) was used for qPCR. The relative target gene expression level was determined in relation to GAPDH using the 2^-ΔCT^ method.

**Table 1:** Summary of PCR/qPCR primer pairs

Name	PCR/ qPCR	Mean product size	Primer	Sequence (5′ to 3′)
MMP9	qPCR	106bp	forward	TTCCAAACCTTTGAGGGCGA
			reverse	CAAAGGCGTCGTCAATCACC
ICAM1	qPCR	149bp	forward	GGTAGCAGCCGCAGTCATAA
			reverse	GATAGGTTCAGGGAGGCGTG
CCL2	PCR	297bp	forward	GCTCAGCCAGATGCAATCAATG
			reverse	GTGTCTGGGGAAAGCTAGGG
CLDN1	qPCR	118bp	forward	CTGTCATTGGGGGTGCGATA
			reverse	CTGGCATTGACTGGGGTCAT
ADAMTS9	PCR	301bp	forward	TTAATCTCACCGCCAATGC
			reverse	GCGCTGCGCCTATAAATGAT
VEGFC	PCR	320bp	forward	CATGTACGAACCGCCAG
			reverse	TTGGCTGTTTGGTCATTGGC
IL4I1	PCR	451bp	forward	TCACCAAGAGCTGGAGACACC
			reverse	AACTTGGTCAGGTTGAGCCC
IL7R	PCR	281bp	forward	TAATAGCTCAGGGGAGATGGAT
			reverse	CTTGCAGAAAACCTTCCACTTCA
TNFAIP3	PCR	629bp	forward	CAACTGAAACGGGGCAAAGC
			reverse	GCCGTCACCGTTCGTTTTC
β-actin	PCR	733bp	forward	CCTCGCCTTTGCCGATCC
			reverse	GGCCATCTCTTGCTCGAAGT
GAPDH	qPCR	87bp	forward	TGCACCACCAACTGCTTAGC
			reverse	GGCATGGACTGTGGTCATGAG

### Western blot

M13SV1-Cre cells and MDA-MB-435-pFDR1 cells were cultivated for 72 h at 37 °C and 5% CO_2_ under normoxic and hypoxic conditions in the presence of TNF-α (100 ng/ml), minocycline (10 μg/ml) and a combination of both. Subsequently, cells were harvested and were lysed in ice-cold RIPA buffer (50 mM Tris-HCl pH 8.0; 150 mM NaCl, 1% (*v*/v) NP-40, 0.5% (*w*/*v*) sodium deoxycholate, 0.1% (w/v) sodium dodecyl sulphate) supplemented with cOmplete, Mini, EDTA-free Protease Inhibitor Cocktail (Sigma Aldrich, Taufkirchen, Germany) and Pierce Phosphatase Inhibitor Mini Tablets (ThermoFisher Scientific GmbH, Schwerte, Germany). The samples were sonicated three times (10 s on and 30 s off) and the total protein concentration was determined using the Pierce™ BCA Protein Assay Kit (ThermoFisher Scientific GmbH, Schwerte, Germany) according to the manufacturer’s instructions. For Western blots, 40 μg of total protein lysate was mixed with 3× Laemmli Sample Buffer and was incubated for 6 min at 95 °C. The samples were separated by 10% or 15% sodium dodecyl sulphate-polyacrylamide gel electrophoresis (SDS-PAGE) and transferred to an Immobilon polyvinyl difluoride (PVDF) nitrocellulose membrane (Merck Millipore, Darmstadt, Germany) under semi-dry conditions. The membranes were blocked in 5% (*w*/*v*) non-fat milk powder in PBS-T (phosphate-buffered saline) with 0.1% (*v*/v) Tween 20 (PBS-T) for 1 h at room temperature. The following antibodies were used for Western blot analysis: anti-MMP9 (rabbit monoclonal; Abcam, Cambridge, UK), anti-CCL2 (MCP-1; rabbit polyclonal; Abcam, Cambridge, UK), anti-claudin-1 (mouse monoclonal; Abcam, Cambridge, UK), anti-ICAM1/CD54 (rabbit polyclonal; Cell Signaling, Leiden, Netherlands), β-actin (rabbit monoclonal; Cell Signaling, Leiden, Netherlands); anti-mouse-IgG-HRP-linked (Cell Signaling, Leiden, Netherlands), and anti-rabbit-IgG-HRP-linked (Cell Signaling, Leiden, Netherlands). The bands were visualized using the Pierce ECL Western blot substrate (Thermo Fisher Scientific, Bonn, Germany), according to the manufacturer’s instructions, and the Aequoria Macroscopic Imaging System (Hamamatsu Photonics Germany, Herrsching am Ammersee, Germany).

### Zymography

The proteolytic activity of MMP9 was measured using a gelatine zymography assay. M13SV1-Cre cells and MDA-MB-435-pFDR1 cells were plated at a density of 5×10^5^ cells/well in a 6-well plate. After a 72 h incubation with 100 ng/ml TNF-α, 10 μg/ml minocycline, and a combination of both, the cell culture supernatants were collected and were mixed with non-reducing Laemmli sample buffer (250 mM Tris-HCl (pH 6.8), 10% (*w*/*v*) SDS, 25% (*v*/v) glycerol, 0.01% (w/v) bromophenol blue) without boiling. The samples were separated on a 10% sodium dodecyl sulphate polyacrylamide gel containing 0.1% gelatine (Sigma Aldrich, Taufkirchen, Germany). After separation by electrophoresis, the gels were washed four times in wash buffer (50 mM Tris-HCl (pH 7.5), 10 mM CaCl_2_, 2.5% (*v*/v) Triton X-100, 0.02% NaN_3_) for 2 h at room temperature to remove the SDS. Then, the gel was incubated in incubation buffer (50 mM Tris-HCl (pH 7.5), 150 mM NaCl, 10 mM CaCl_2_, 0.02% NaN_3_) overnight at 37 °C. Subsequently, the gel was stained with Coomassie brilliant blue R-250 (Sigma Aldrich, Taufkirchen, Germany) for 1 h at room temperature with gentle agitation. Finally, the gels were destained until clear bands appeared visible, which were indicative of the proteolytic activity of proteases, including MMP9.

### XTT assay

To determine the appropriate minocycline concentration for the quantification of cell fusions, M13SV1-Cre and MDA-MB-435-pFDR1 cells were cultured in triplicate at a ratio of 1:3 for up to 3 days in the presence of different concentrations of minocycline. Non-treated cells served as a control. After 24 h, 48 h, and 72 h the media was removed and the plates were analysed using the XTT reagent (Roche Diagnostics, Mannheim, Germany) according to the manufacturer’s instructions. Absorption of the XTT-formazan derivative that formed was measured using a BioTek EL800 microplate reader (BioTek, Bad Friedrichshall, Germany).

### siRNA experiments

Expression of claudin-1 was knocked-down using a specific claudin-1 siRNA (Santa Cruz Biotechnology, Heidelberg, Germany). For control purposes, a non-targeting negative control siRNA was used (QIAGEN GmbH, Hilden, Germany). M13SV1-Cre cells (2.5×10^5^) were resuspended in Opti-Mem (ThermoFisher Scientific, Schwerte, Germany) and were subsequently transfected with claudin-1 siRNA or control siRNA to a final concentration of 100 nM by lipofection (Lipofectamine™ 2000; ThermoFisher Scientific, Schwerte, Germany) as described in the user’s manual. The cells were seeded in serum-free MSU medium in 6-well plates. After 6 h, the serum-free media was replaced with complete MSU medium containing FCS, antibiotics and additional supplements. The knock-down of claudin-1 expression in M13SV1-Cre cells was confirmed by Western blot.

### Co-immunoprecipitation

M13SV1-Cre cells and MDA-MB-435-pFDR1 cells were stimulated with 100 ng/ml TNF-α, 10 μg/ml minocycline, or a combination of both, for 72 h at 37 °C and 5% CO_2_ in a humidified atmosphere. Subsequently, the cells were washed once with PBS and lysed in ice-cold lysis buffer (1% (*v*/*v*) NP-40, 50 mM Tris-HCl (pH 7.5), 150 mM NaCl, 1 mM EDTA, 1 mM EGTA and proteinase inhibitors (see above)) for 30 min on ice. The samples were sonicated three times (5 s on/20 s off) on a low pulse frequency and the total protein concentration was determined using the Pierce™ BCA Protein Assay Kit (ThermoFisher Scientific GmbH, Schwerte, Germany) according to the manufacturer’s instructions. The lysates were cleared by centrifugation (10 min, 12,000 rpm) and the supernatants were transferred to a new tube. Prior to immunoprecipitation, the lysates were pre-treated with 25 μl of Protein A Magnetic Beads (Cell Signaling, Leiden, Netherlands) for 2 h at 4 °C to remove any proteins that might bind non-specifically to Protein A. The samples were incubated with anti-ICAM1/CD54 (5 μg/ml; mouse monoclonal, clone 11C81; Bio-Techne GmbH, Wiesbaden-Nordenstadt, Germany) or IgG1 (5 μg/ml; Beckman Coulter, Krefeld, Germany) overnight at 4 °C. Thereafter, 30 μl of Protein A Magnetic Beads were added and the samples were incubated for an additional 2 h at 4 °C. The precipitates were washed three times in lysis buffer, separated on a magnetic rack and resuspended in 3× Laemmli Sample Buffer (with DTT, without β-mercaptoethanol). ICAM1/CD54 and the co-immunoprecipitated proteins were detected by Western blot.

### Soluble ICAM1 detection

To detect soluble ICAM1, the cell culture supernatants from TNF-α-treated, minocycline-treated, and TNF-α and minocycline-treated M13SV1-Cre cells and MDA-MB-435-pFDR1 cells were collected and concentrated using Vivaspin-2 centrifugal concentrators (100,000 MWCO; Sartorius, Göttingen, Germany). The total protein concentration of the concentrated supernatants was determined using the Pierce™ BCA Protein Assay Kit (ThermoFisher Scientific GmbH, Schwerte, Germany) according to the manufacturer’s instructions. An equal amount of supernatant from each sample was resuspended in Laemmli sample buffer. Soluble ICAM1 was detected by Western blot using an anti-ICAM1/CD54 antibody (clone 14C11, mouse monoclonal; ThermoFisher Scientific GmbH, Schwerte, Germany).

### Statistical analyses

The statistical significance of the data presented in Fig. 2 was calculated using an unpaired, two-tailed Student’s *t*-test. The mean differences of the data presented in Figs. 4, 5, 6 and 7 were tested by ANOVA F-tests. Afterwards, multiple comparisons were performed using Scheffé post-hoc tests. Statistical analyses were performed using SPSS Version 23.0.0.2 and *p*-values < 0.05 were considered significant.

### Language editing

The manuscript was language edited by American Journal Experts (Durham, NC, USA).

## Results

### Analysis of the gene expression pattern of M13SV1-Cre cells and MDA-MB-435-pFDR1 cells with TNF-α treatment in normoxic and hypoxic conditions

**Fig. 1:**
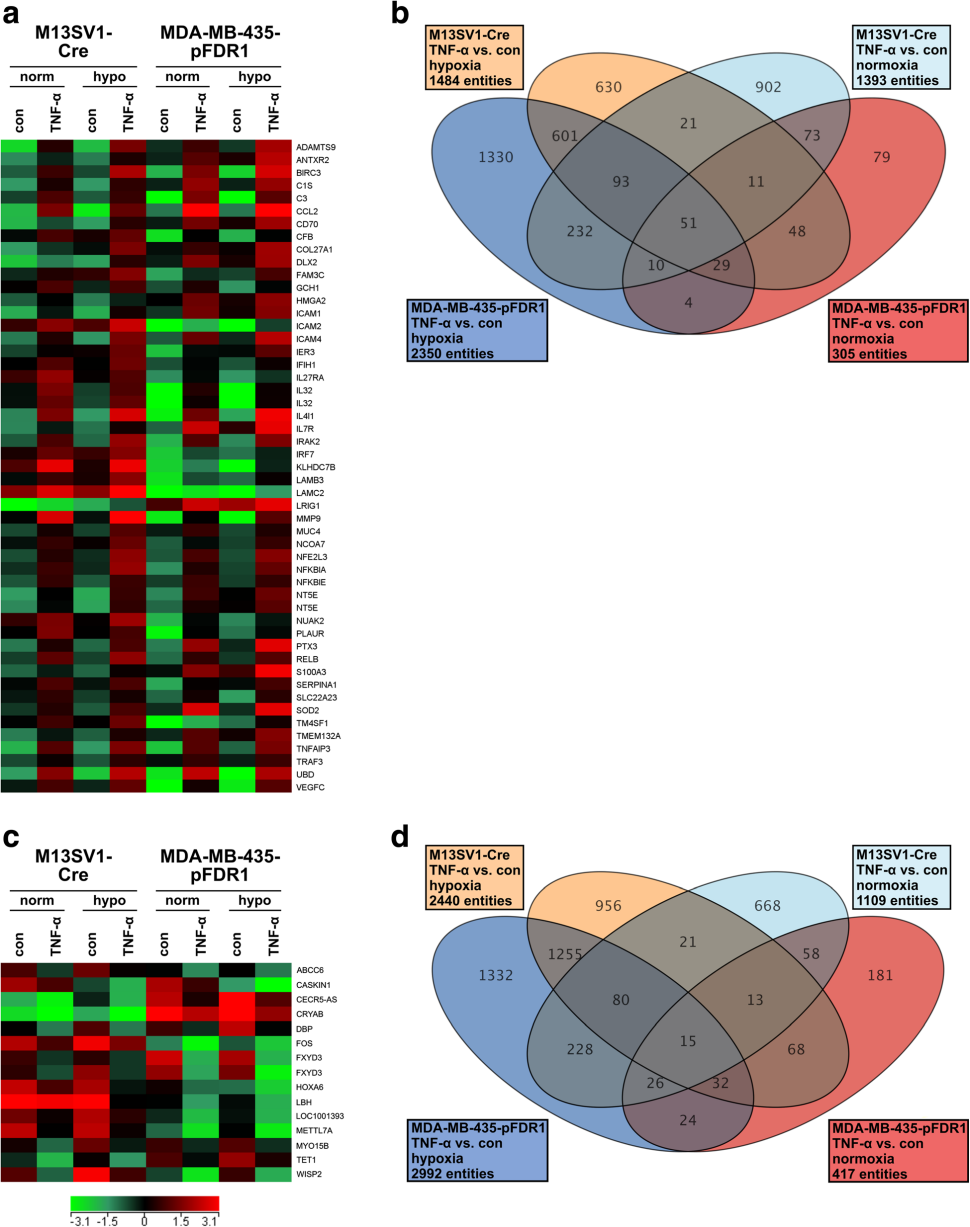
Differentially regulated genes in TNF-α-treated M13SV1-Cre and MDA-MB-435-pFDR1 cells under normoxic and hypoxic conditions. The up-regulated and down-regulated genes in TNF-α-treated cells compared with untreated cells that matched the filter criteria (flags: not detected flags and compromised spots were removed) and fold changes (2-fold up and down). **a** A heat map analysis of TNF-α up-regulated genes, **b** a Venn diagram of TNF-α up-regulated genes, **c** a heat map analysis of TNF-α down-regulated genes, **d** a Venn diagram of TNF-α down-regulated genes

We recently demonstrated that the fusion of human M13SV1-Cre breast epithelial cells and human MDA-MB-435-pFDR1 breast cancer cells under normoxic and hypoxic conditions is positively triggered by TNF-α [18]. To analyse the changes in the gene expression profile of both cell lines induced by TNF-α (100 ng/ml) under normoxic and hypoxic conditions, cDNA-microarray studies were performed. In total, 51 genes were found to be significantly up-regulated in TNF-α-treated cell lines under both normoxia and hypoxia (Fig. 1a, b; Table 2), including adhesion molecules such as ICAM1, ICAM2, and ICAM4, components of the extracellular matrix, including COL27A1, LAMB3, LAMC2, and MUC4, chemokines, growth factors, and interleukins, such as CCL2, VEGFC and IL32, and proteases including ADAMTS9 and MMP9 (Fig. 1; Table 2). In particular, CCL2, MMP9 and ICAM1 have already been associated with cell fusion [9, 28]. In contrast, 15 genes were down-regulated in TNF-α-treated cells under normoxic and hypoxic conditions, such as ABCC6 and FOS (Fig. 1c, d; Table 3).

**Table 2:** Up-regulated genes (FC ≥ 2) in TNF-α treated cells under normoxic and hypoxic conditions

			Normoxia	Hypoxia	Normoxia	Hypoxia
Genbank Accession	GeneSymbol	GeneName	M13SV1-Cre + TNF-α vs. M13SV1-Cre	M13SV1-Cre + TNF-α vs. M13SV1-Cre	MDA-MB-435-pFDR1 + TNF-α vs. MDA-MB-435-pFDR1	MDA-MB-435-pFDR1 + TNF-α vs. MDA-MB-435-pFDR1
NM_182920	ADAMTS9	ADAM metallopeptidase with thrombospondin type 1 motif, 9	15.34	25.45	3.05	10.59
NM_058172	ANTXR2	anthrax toxin receptor 2	2.64	4.43	3.61	5.63
NM_001165	BIRC3	baculoviral IAP repeat containing 3	4.55	13.11	23.74	97.65
NM_001734	C1S	complement component 1, s subcomponent	5.66	6.97	6.91	7.03
NM_000064	C3	complement component 3	4.31	4.24	182.13	115.58
NM_002982	CCL2	chemokine (C-C motif) ligand 2	25.36	39.20	179.73	62.92
NM_001252	CD70	CD70 molecule	3.15	6.99	4.76	4.52
NM_001710	CFB	complement factor B	2.41	2.28	11.21	5.74
NM_032888	COL27A1	collagen, type XXVII, alpha 1	2.70	4.45	2.09	5.29
NM_004405	DLX2	distal-less homeobox 2	2.39	7.50	5.38	4.42
NM_014888	FAM3C	family with sequence similarity 3. member C	2.08	2.50	3.27	4.06
NM_001024071	GCH1	GTP cyclohydrolase 1	2.20	3.07	2.93	3.83
NM_001300919	HMGA2	high mobility group AT-hook 2	2.26	2.35	3.27	3.66
NM_000201	ICAM1	intercellular adhesion molecule 1	4.46	6.91	5.76	3.58
NM_000873	ICAM2	intercellular adhesion molecule 2	2.53	4.04	4.71	46.87
NM_022377	ICAM4	intercellular adhesion molecule 4 (Landsteiner-Wiener blood group)	4.47	15.53	7.15	11.04
NM_003897	IER3	immediate early response 3	2.02	2.84	6.61	2.96
NM_022168	IFIH1	interferon induced with helicase C domain 1	2.43	3.47	2.01	2.48
NM_004843	IL27RA	interleukin 27 receptor. Alpha	2.65	2.31	3.47	2.56
NM_001012633	IL32	interleukin 32	4.47	4.23	45.05	61.04
NM_001012631	IL32	interleukin 32	3.53	5.65	174.41	103.38
NM_152899	IL4I1	interleukin 4 induced 1	13.76	46.63	50.44	74.89
NM_002185	IL7R	interleukin 7 receptor	2.81	3.65	23.52	8.37
NM_001570	IRAK2	interleukin-1 receptor-associated kinase 2	5.28	13.66	14.76	14.90
NM_004031	IRF7	interferon regulatory factor 7	2.30	2.31	4.20	2.17
NM_138433	KLHDC7B	kelch domain containing 7B	5.74	10.45	2.77	13.77
NM_001017402	LAMB3	laminin, beta 3	2.12	3.48	5.68	2.89
NM_005562	LAMC2	laminin, gamma 2	2.73	4.65	2.05	4.42
NM_015541	LRIG1	leucine-rich repeats and immunoglobulin-like domains 1	2.29	2.49	4.45	2.27
NM_004994	MMP9	matrix metallopeptidase 9 (gelatinase B, 92 kDa gelatinase, 92 kDa type IV collagenase)	11.45	27.25	13.49	44.92
NM_018406	MUC4	mucin 4, cell surface associated	2.49	4.72	2.34	2.76
NM_181782	NCOA7	nuclear receptor coactivator 7	2.08	2.89	2.14	3.31
NM_004289	NFE2L3	nuclear factor. Erythroid 2-like 3	3.08	7.27	5.00	8.99
NM_020529	NFKBIA	nuclear factor of kappa light polypeptide gene enhancer in B-cells inhibitor, alpha	2.97	7.63	5.41	5.74
NM_004556	NFKBIE	nuclear factor of kappa light polypeptide gene enhancer in B-cells inhibitor, epsilon	3.86	4.40	3.41	3.14
NM_002526	NT5E	5′-nucleotidase, ecto (CD73)	4.37	9.64	7.12	3.26
NM_002526	NT5E	5′-nucleotidase, ecto (CD73)	3.37	8.28	3.62	2.54
NM_030952	NUAK2	NUAK family, SNF1-like kinase. 2	2.24	5.48	5.71	2.87
NM_001005377	PLAUR	plasminogen activator, urokinase receptor	4.00	2.12	14.94	2.74
NM_002852	PTX3	pentraxin 3, long	4.37	6.10	16.59	17.75
NM_006509	RELB	v-rel avian reticuloendotheliosis viral oncogene homolog B	5.02	8.99	4.61	4.41
NM_002960	S100A3	S100 calcium binding protein A3	2.01	2.88	4.90	7.84
NM_001002236	SERPINA1	serpin peptidase inhibitor, clade A (alpha-1 antiproteinase. antitrypsin), member 1	2.50	2.73	5.44	2.35
NM_015482	SLC22A23	solute carrier family 22, member 23	2.08	2.23	3.21	7.09
NM_001024465	SOD2	superoxide dismutase 2, mitochondrial	2.48	5.20	16.52	19.28
NM_014220	TM4SF1	transmembrane 4 L six family member 1	2.08	3.23	5.34	3.43
NM_017870	TMEM132A	transmembrane protein 132A	2.63	3.35	3.74	3.53
NM_006290	TNFAIP3	tumor necrosis factor, alpha-induced protein 3	14.81	16.94	19.45	9.77
NM_145725	TRAF3	TNF receptor-associated factor 3	2.21	3.61	2.02	2.50
NM_006398	UBD	ubiquitin D	19.59	58.56	100.44	226.21
NM_005429	VEGFC	vascular endothelial growth factor C	2.76	4.16	74.91	33.90

**Table 3:** Down-regulated genes (FC ≤ −2) in TNF-α treated cells under normoxic and hypoxic conditions

			Normoxia	Hypoxia	Normoxia	Hypoxia
Genbank Accession	GeneSymbol	GeneName	M13SV1-Cre + TNF-α vs. M13SV1-Cre	M13SV1-Cre + TNF-α vs. M13SV1-Cre	MDA-MB-435-pFDR1 + TNF-α vs. MDA-MB-435-pFDR1	MDA-MB-435-pFDR1 + TNF-α vs. MDA-MB-435-pFDR1
NM_001079528	ABCC6	ATP-binding cassette, sub-family C (CFTR/MRP), member 6	−2.72	−2.26	−2.51	−2.36
NM_020764	CASKIN1	CASK interacting protein 1	−2.03	−2.26	−2.52	−7.21
NR_024482	CECR5-AS1	CECR5 antisense RNA 1	−2.10	− 2.94	−3.66	−6.97
NM_001885	CRYAB	crystallin, alpha B	−2.76	−4.62	−2.03	−6.23
NM_001352	DBP	D site of albumin promoter (albumin D-box) binding protein	−2.36	−4.44	−2.20	−4.92
NM_005252	FOS	FBJ murine osteosarcoma viral oncogene homolog	−2.45	−2.72	−3.24	− 2.60
NM_001136007	FXYD3	FXYD domain containing ion transport regulator 3	−2.32	−2.06	−19.31	−14.74
NM_001136008	FXYD3	FXYD domain containing ion transport regulator 3	−2.44	−4.64	−10.82	−19.67
XM_006715716	HOXA6	homeobox A6	−2.75	−4.61	−2.38	− 2.30
NM_030915	LBH	limb bud and heart development	−2.03	−31.03	−2.93	−3.35
NR_024485	LOC100130093	uncharacterized LOC100130093	−2.28	−3.05	−3.11	− 3.30
NM_014033	METTL7A	methyltransferase like 7A	−4.74	−4.34	−2.93	−6.66
BC128044	MYO15B	myosin XVB pseudogene	−2.42	−2.81	− 2.15	−2.70
NM_030625	TET1	tet methylcytosine dioxygenase 1	−2.90	−3.02	−2.01	− 2.70
NM_003881	WISP2	WNT1 inducible signaling pathway protein 2	−3.96	−4.69	−3.99	−5.53

### Validation of microarray data by qPCR and conventional PCR

**Fig. 2:**
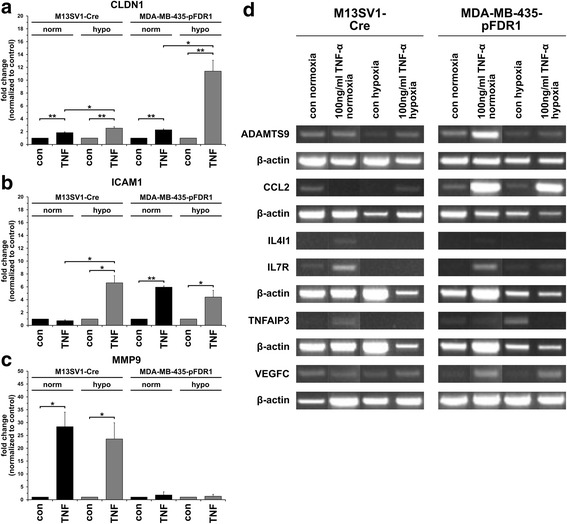
Validation of microarray data by qPCR and conventional PCR. **a** qPCR CLDN1, **b** qPCR ICAM1, **c** qPCR MMP9, **d** conventional PCR. MMP9 was significantly up-regulated in TNF-α-treated (100 ng/ml) M13SV1-Cre cells, whereas significantly elevated CLDN1 and ICAM1 expression levels were detected in TNF-α-treated (100 ng/ml) MDA-MB-435-pFDR1 cells. Likewise, increased CCL2 and VEGFC expression levels were detected in TNF-α-treated (100 ng/ml) MDA-MB-435-pFDR1 cells. Data are presented as the mean ± SD of at least three independent experiments (**a**) or the representative data of three independent experiments (**b**). Statistical analysis: unpaired, two-tailed Student’s *t*-test: * = *p* < 0.05; ** = *p* < 0.01

To validate the microarray data, Claudin-1 (CLDN1), ICAM1, and MMP9 expression was analysed by qPCR and ADAMTS9, CCL2, IL4I1, IL7R, TNFAIP3 and VEGFC expression was analysed by conventional PCR. CLDN1 did not pass the filter criteria but the microarray data revealed a marked up-regulation of this protein in TNF-α-treated (100 ng/ml) MDA-MB-435-pFDR1 cells under normoxic and hypoxic conditions (Additional file 1). The qPCR and conventional PCR data partially matched the microarray data (Fig. 2a, d). The microarray data revealed an up-regulation of ICAM1 expression in TNF-α-stimulated (100 ng/ml) M13SV1-Cre cells under normoxic conditions (Fig. 1; Table 2), which was opposite to the qPCR data showing similar ICAM1 mRNA levels in untreated and TNF-α-treated M13SV1-Cre cells under normoxic conditions (Fig. 2b). In contrast, ICAM1 was up-regulated in TNF-α-stimulated M13SV1-Cre cells under hypoxia (Fig. 1; Table 2), which could be validated by qPCR (Fig. 2b). The TNF-α-induced increase in CLDN1 and MMP9 expression in M13SV1-Cre cells under normoxia and hypoxia (Fig. 1; Table 2) were validated by qPCR, whereas MMP9 mRNA expression was markedly up-regulated in TNF-α-stimulated cells (Fig. 2c). For MDA-MB-435-pFDR1 cells, only the microarray data for CLDN1 and ICAM1 could be validated by qPCR; the qPCR data for MMP9 did not match the microarray data (Figs. 1, 2c; Table 2). Markedly increased MMP9 expression levels were determined for TNF-α-stimulated MDA-MB-435-pFDR1 cells (Fig. 1; Table 2), which is opposite to qPCR rather showing comparable MMP9 mRNA expression levels in untreated and TNF-α treated cells (Fig. 2c).

CCL2, IL7R1, and VEGFC expression levels were partially up-regulated in TNF-α-stimulated MDA-MB-435-pFDR1 cells, which agreed with the microarray data (Fig. 1, 2b; Table 2). In contrast, the microarray data revealed an up-regulation of IL4I1 in TNF-α-treated MDA-MB-435-pFDR1 cells, which could not be validated by conventional PCR (Fig. 2d). Instead, only a weak PCR product was observed in TNF-α-stimulated MDA-MB-435-pFDR1 cells under normoxic conditions, whereas under hypoxia no PCR product was detected (Fig. 2d). TNF-α induced an increase in IL4I1, IL7R and TNFAIP3 expression in M13SV1-Cre cells under normoxic conditions (Fig. 2d) and in hypoxic conditions there was only a slight increase in VEGFC levels in response to TNF-α treatment (Fig. 2d). Hence, the M13SV1-Cre microarray data were only partially validated by conventional PCR (Figs. 1, 2d; Table 2).

### Validation of the microarray data by western blot

**Fig. 3:**
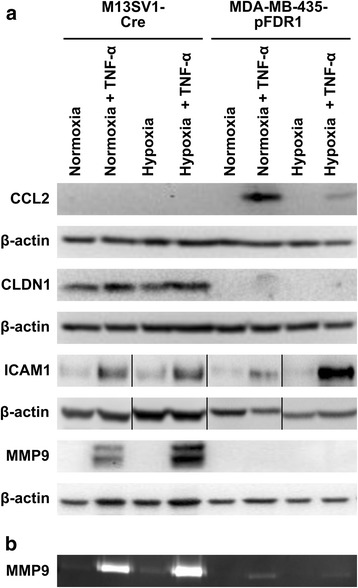
Validation of qPCR and conventional RT-PCR data by Western blot and zymography. **a** Western blot analysis: CCL2 protein expression was only detected in TNF-α-treated (100 ng/ml) MDA-MB-435-pFDR1 cells, whereas CLDN1 expression was absent from these cells but was clearly detectable in M13SV1-Cre cells. Increased ICAM1 expression was observed in TNF-α-treated cells, whereas increased MMP9 expression was only found in TNF-α-stimulated M13SV1-Cre cells. Two discrete bands were detected for MMP9 in Western blot analysis; the upper band represents inactive MMP9 and the lower band represents active MMP9. ICAM1 Western blot data were rearranged because ICAM1 samples were originally loaded in a different order. **b** Zymography: markedly increased MMP9 expression levels and activity were detected in TNF-α-treated M13SV1-Cre cells, whereas even in TNF-α-stimulated MDA-MB-435 cells slightly enhanced MMP9 expression levels could be identified. The data shown are representative of at least three independent experiments

To further validate the microarray and PCR data, Western blot analyses were conducted for CCL2, CLDN1, ICAM1 and MMP9. TNF-α-induced (100 ng/ml) CCL2 expression was observed in MDA-MB-435-pFDR1 cells with higher CCL2 expression under normoxia compared with hypoxia (Fig. 3a). In contrast, no CCL2 expression was found in M13SV1-Cre human breast epithelial cells (Fig. 3a), which agrees with conventional PCR data (Fig. 2b). The expression of CLDN1 was only observed in M13SV1-Cre cells concomitant with slightly increased CLDN1 levels in the presence of TNF-α (Fig. 3a), which is similar to qPCR data (Fig. 2). Interestingly, no CLDN1 protein expression was detected in MDA-MB-435-pFDR1 cells (Fig. 3a) even though qPCR data revealed increased CLDN1 mRNA levels in TNF-α-treated cells (Fig. 2b). In agreement with the microarray data (Fig. 1; Table 2), but opposite to the qPCR data (Fig. 2a), increased ICAM1 protein expression was identified in TNF-α-treated M13SV1-Cre cells (Fig. 3a). Increased TNF-α-induced MMP9 levels were only observed in M13SV1-Cre cells (Fig. 3a), which agreed with the qPCR data (Fig. 2c). In addition to Western blot analysis, MMP9 expression was further analysed by zymography, which also showed increased MMP9 expression in TNF-α-treated M13SV1-Cre cells (Fig. 3b). Zymography analysis revealed a slight up-regulation of MMP9 expression in TNF-α-stimulated MDA-MB-435-pFDR1 cells (Fig. 3b), which confirmed the qPCR data showing a weak MMP9 PCR product in TNF-α-treated cells under normoxic conditions (Fig. 2c).

### Fusion of M13SV1-Cre cells and MDA-MB-435-pFDR1 cells is impaired by the inhibition of MMP9

**Fig. 4:**
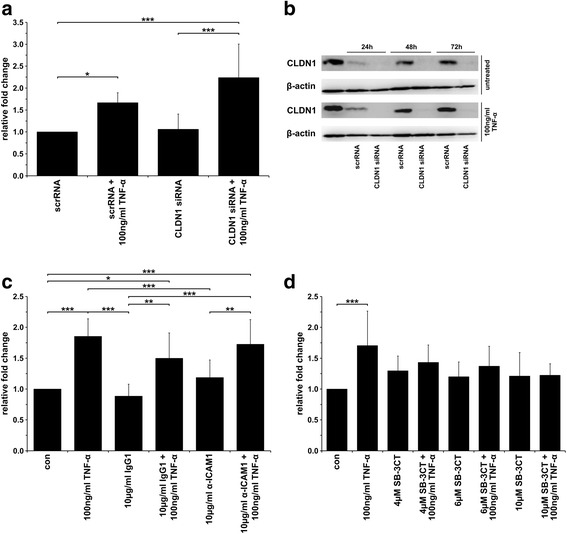
TNF-α-induced cell fusion could be blocked by the inhibition of MMP9. **a** The knockdown of CLDN1 expression with specific siRNA was not correlated with a decreased TNF-α-induced (100 ng/ml) fusion rate. **b** siRNA-mediated CLDN1 expression levels of M13SV1-Cre cells were stably down-regulated over 72 h. **c** Inhibition of ICAM1 activity using a blocking antibody did not impair the TNF-α-induced fusion between M13SV1-Cre and MDA-MB-435-pFDR1 cells. **d** The TNF-α-induced fusion of M13SV1-Cre and MDA-MB-435-pFDR1 cells was impaired by the MMP9 inhibitor SB-3CT in a dose-dependent manner. The mean ± SD of at least three independent experiments is shown. Statistical analysis: ANOVA F-test and Scheffé post-hoc test: * = *p* < 0.05; ** = *p* < 0.01; *** = *p* < 0.001

To investigate whether CLDN1, ICAM1 and MMP9 might be involved in the TNF-α-induced fusion of M13SV1-Cre and MDA-MB-435-pFDR1 cells, the expression of these proteins was blocked. The knockdown of CLDN1 with siRNA in M13SV1-Cre cells did not impair the TNF-α-induced fusion of the cells (Fig. 4a) even though the siRNA-mediated down-regulation of CLDN1 expression in M13SV1-Cre was stable for at least 3 days (Fig. 4b). Interestingly, compared with M13SV1-Cre cells that were transfected with scrambled siRNA, M13SV1-Cre cells transfected with CLDN1 siRNA had a slightly increased fusion rate (Fig. 4a).

The inhibition of ICAM1 function with a blocking ICAM1 antibody was not correlated with a decreased TNF-α-induced rate of the fusion of M13SV1-Cre and MDA-MB-435-pFDR1 cells (Fig. 4c). In contrast, the inhibition of MMP9 function using the specific inhibitor SB-3CT was associated with a dose-dependent, impaired, TNF-α-induced cell fusion rate of M13SV1-Cre and MDA-MB-435-pFDR1 cells (Fig. 4d).

### The TNF-α-induced fusion of M13SV1-Cre and MDA-MB-435-pFDR1 cells is inhibited by minocycline

The tetracycline based antibiotic minocycline was tested because of its capability to down-regulate MMP9 expression in cells [29, 30]. First, XTT proliferation studies were performed to determine the optimal minocycline concentration and a range between 5 μg/ml and 24 μg/ml was tested. Prolonged cultivation of M13SV1-Cre and MDA-MB-435-pFDR1 cells with increasing concentrations of minocycline was correlated to a decreased cell proliferation rate (Additional file 2). Hence, for on-going studies, a minocycline concentration of 10 μg/ml was chosen and only the impact of minocycline under normoxic conditions was analysed. The cultivation of cells under hypoxia and in the presence of 10 μg/ml minocycline was associated with numbers of dead cells that were too high to perform further cell fusion quantification studies.

**Fig. 5:**
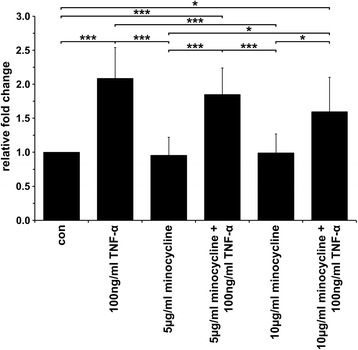
TNF-α-induced fusion was inhibited by the tetracycline-based antibiotic minocycline. TNF-α-induced (100 ng/ml) cell fusion was impaired by minocycline in a dose-dependent manner. The data shown are the mean ± SD of three independent experiments. Statistical analysis: ANOVA F-test and Scheffé post-hoc test: * = *p* < 0.05; ** = *p* < 0.01; *** = *p* < 0.001

The incubation of cells with 5 μg/ml or 10 μg/ml minocycline was correlated to a dose-dependent decreased TNF-α-induced fusion rate (Fig. 5), whereas the spontaneous fusion rate of the cells remained unaffected in the presence of different minocycline concentrations (Fig. 5).

### Effect of minocycline on CLDN1, ICAM1, and MMP9 expression

**Fig. 6:**
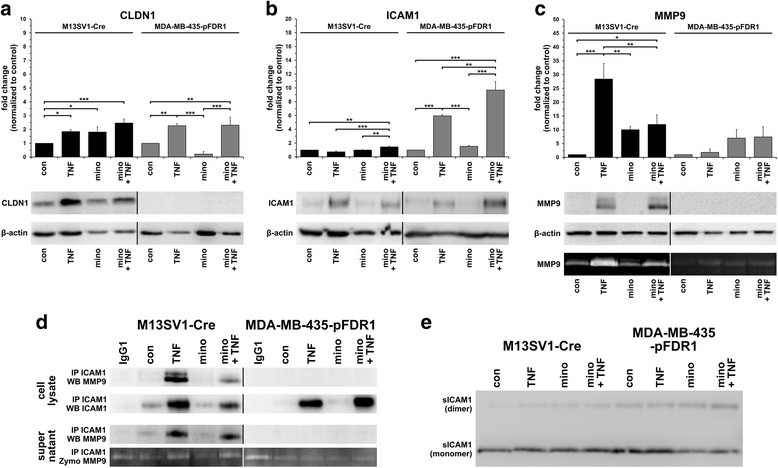
TNF-α-induced MMP9 expression in M13SV1-Cre cells was blocked by minocycline. M13SV1-Cre and MDA-MB-435-pFDR1 cells were treated for 3 days with 100 ng/ml TNF-α, 10 μg/ml minocycline and a combination of both. The expression levels of **a**) CLDN1, **b**) ICAM1 and **c**) MMP9 were determined by qPCR, Western blot analysis and zymography (only MMP9). Significantly decreased MMP9 mRNA levels were detected in TNF-α and minocycline-treated M13SV1-Cre cells as compared with TNF-α-treated cells and were validated by zymography. **d**) Co-immunoprecipitation data show that MMP9 binds to ICAM1. ICAM1 expression and co-immunoprecipitated MMP9 were markedly decreased in the lysates of TNF-α + minocycline-treated M13SV1-Cre cells compared with the lysates from TNF-α-stimulated cells. **e**) No differences in sICAM1 levels were detected in M13SV1-Cre and MDA-MB-435-pFDR1 cells that were treated with TNF-α, minocycline and a combination of both. Shown are the mean ± SD or representative Western blot data for at least three independent experiments. Statistical analysis: ANOVA F-test and Scheffé post-hoc test: * = *p* < 0.05; ** = *p* < 0.01; *** = *p* < 0.001

Quantitative PCR and Western blot data showed that CLDN1 and ICAM1 expression in TNF-α-treated and TNF-α and minocycline-treated M13SV1-Cre cells were comparable (Fig. 6a, b). In contrast, the qPCR results showed significantly reduced MMP9 expression in M13SV1-Cre cells co-treated with TNF-α and minocycline as compared with TNF-α-treated cells (Fig. 6c), which was further validated by zymography (Fig. 6c). MMP9 protein expression was slightly increased in TNF-α + minocycline co-treated M13SV1-Cre cells compared with TNF-α-stimulated cells (Fig. 6c). This (reproducible) result remains ambiguous as did the finding that significantly higher MMP9 mRNA levels were observed in minocycline treated M13SV1-Cre cells (Fig. 6c) although increased MMP9 protein expression levels were not detected (Fig. 6c).

In contrast to M13SV1-Cre breast epithelial cells, neither CLDN1 nor MMP9 protein expression was detected in MDA-MB-435-pFDR1 cells (Fig. 6a, c). Because very weak MMP9 bands were observed in MDA-MB-435-pFDR1 cells by zymography (Fig. 6c), and MMP9 and CLDN1 were detected by qPCR (Fig. 6a, c) we assume that MMP9 and CLDN1 protein expression levels were below the detection threshold of the antibodies that were used. Minocycline treatment increased MMP9 mRNA expression in both M13SV1-Cre cells and MDA-MB-435-pFDR1 cells (Fig. 6c), which, however, did not get translated into increased expression of the subsequent proteins (Fig. 6c). Compared with M13SV1-Cre breast epithelial cells, increased ICAM1 expression levels were found in TNF-α- and TNF-α + minocycline-treated MDA-MB-435-pFDR1 cells (Fig. 6b). Interestingly, significantly higher ICAM1 mRNA and protein levels were detected in the TNF-α + minocycline-treated MDA-MB-435-pFDR1 cells than in the TNF-α-stimulated cells (Fig. 6b).

Co-immunoprecipitation assays were performed to investigate whether MMP9 bound to ICAM1 and whether this would be correlated to increased soluble ICAM1 (sICAM1) levels because of MMP9-dependent proteolytic degradation [31]. As shown in Fig. 6d, MMP9 could be co-immunoprecipitated with ICAM1 in M13SV1-Cre cells but not in MDA-MB-435-pFDR1 cells. Higher ICAM1 and MMP9 levels were co-immunoprecipitated in TNF-α-stimulated M13SV1-Cre cells, whereas lower levels were detected in cells co-treated with TNF-α and minocycline (Fig. 6d), which is in agreement with the Western blot data (Fig. 6c). In contrast, because of rather low MMP9 expression levels in MDA-MB-435-pFDR1 cells, MMP9 was not co-immunoprecipitated. To prove, whether the bindings of ICAM1 were proteolytically degraded by MMP9, the supernatants from cell culture were collected. Western blot analysis revealed no differences in the relative amount of sICAM1 monomers and dimers between the control cells and those treated with TNF-α or TNF-α + minocycline.

### Both minocycline and the MMP9 inhibitor SB-3CT impaired TNF-α-mediated cell fusion

**Fig. 7:**
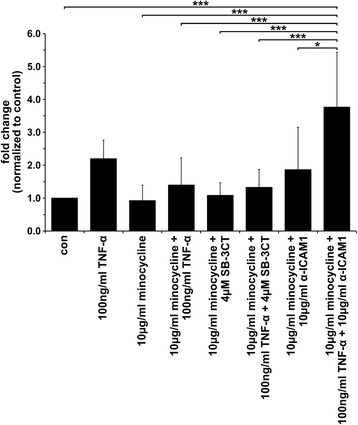
The co-administration of minocycline, SB-3CT and the ICAM1 blocking antibody, yielded different results. No additive inhibitory effect of minocycline and SB-3CT regarding the TNF-α-induced fusion of M13SV1-Cre and MDA-MB-435-pFDR1 cells was observed. In contrast, the incubation of cells in the presence of minocycline, ICAM1 blocking antibody and TNF-α yielded a significantly increased fusion rate. The data shown are the mean ± SD of at least three independent experiments. Statistical analysis: ANOVA F-test and Scheffé post-hoc test: * = *p* < 0.05; ** = *p* < 0.01; *** = *p* < 0.001

The fusion rate of cells co-treated with both minocycline and SB-3CT (and TNF-α) was comparable to that of cells treated with minocycline and minocycline + TNF-α, respectively, and no additive effect was observed (Fig. 7). Interestingly, the cultivation of cells in the presence of both minocycline and the blocking ICAM1 antibody yielded in an increased cell fusion rate, which was more intense in the presence of TNF-α (Fig. 7). Even though the data were not significant, the findings were reproducible in independent experiments, indicating that the increased fusion rate of cells in the presence of minocycline and anti-ICAM1 (and TNF-α) is a true effect.

## Discussion

In a previous study we demonstrated that the pro-inflammatory cytokine TNF-α is a potent trigger of cell fusion between human M13SV1-Cre breast epithelial cells and human breast cancer cell lines under both normoxic and hypoxic conditions [18]. Here, we investigated the impact of TNF-α on the gene expression profiles of M13SV1-Cre and MDA-MB-435-pFDR1 cells to identify which of the differentially-regulated genes might be involved in cell fusion. Of the identified target genes, only the inhibition of MMP9 was correlated with a decreased TNF-α-induced cell fusion rate.

Here, we used the MDA-MB-435 cancer cell line, whose origin is debated to be either breast cancer or melanoma. A systematic analysis of the gene expression profiles of 60 human cancer cell lines first revealed that MDA-MB-435 cells express genes that are highly expressed in most melanoma-derived cell lines, suggesting that this cell line originated from melanoma rather than breast cancer [32]. This assumption was further supported by comparative genomic hybridisation (VGH) and microsatellite polymorphism analyses showing that MDA-MB-435 cells and M14 cells share similarities [33]. In contrast, MDA-MB-435 and M14 cells markedly differed in their DNA methylation profiles; more hypermethylated CpG islands were detected in MDA-MB-435 cells than in M14 cells and MDA-MB-435 cells were not grouped with melanoma cells after a hierarchical cluster analysis [34]. Likewise, the phenotypic and molecular characterisation of MDA-MB-435 cells further revealed similarities to other breast cancer cell lines, such as MDA-MB-231, SUM1315 or HBL100 cells, which belong to the claudin-low intrinsic subtype of breast cancer [35].

PCR data showed that the microphthalmia-associated transcription factor (MITF), which is a lineage survival oncogene amplified in malignant melanoma cell lines, was expressed in MDA-MB-435 cells [36, 37]. However, MITF protein was not expressed in MDA-MB-435 cells [38] but mRNA expression was detected in other breast cancer cell lines, such as MCF-10A, MCF-7, SKBR3, and U87 and D54 glioblastoma cell lines [37]. The co-expression of neuronal, epithelial and melanocytic markers in breast cancer, melanoma and glioblastoma cell lines might be related to lineage infidelity, which seems to be a common phenomenon in cancer cells lines [37]. In agreement with MCF-7 breast cancer cells, but in contrast to melanoma cells, the expression of breast epithelium-specific and epithelial-specific markers, such as β-casein, α-lactalbumin, epithelial membrane antigen (EMA), and keratin-19 as well as the induction of β-casein expression and production of milk lipids by β-heregulin and vitamin E was clearly observed in MDA-MB-435 cells [38]. Moreover, MDA-MB-435 xenograft studies revealed that the vast majority of the cells were positive for cytokeratin and EMA, suggesting that MDA-MB-435 is a breast epithelial cell line that has gained melanocyte lineage characteristics [38].

Our data indicate that the TNF-α-induced fusion of M13SV1-Cre and MDA-MB-435-pFDR1 cells depends on MMP9. Blocking MMP9 with a specific inhibitor or the inhibition of MMP9 expression using minocycline was associated with a decreased cell fusion frequency. These data agree with that of several studies that have already demonstrated that TNF-α and/or MMP9 play a role in the fusion of different cell types [16–19, 39–41]. For instance, Song et al. showed that TNF-α enhanced the fusion of oral squamous carcinoma cells and endothelial cells via a VCAM-1/VLA-4-dependent pathway [19]. MMP9 was not investigated in this particular study and it is unclear whether it is involved in the fusion of these cells. TNF-α-induced VCAM-1 expression in endothelial cells is crucial for close cell-cell contact and is a prerequisite for the subsequent plasma membrane fusion step. Here, TNF-α induced ICAM1 expression in both cell types, but no reduced cell fusion rate was observed in the presence of an ICAM1 blocking antibody. It is likely indicating that the TNF-α-induced fusion of M13SV1-Cre and MDA-MB-435-pFDR1 was independent of ICAM1. Nonetheless, ICAM1 was recently identified to augment myoblast adhesion and fusion through homophilic trans-interactions and Rac-mediated actin remodelling [28]. Therefore, the fusogenic property of ICAM1-ICAM1 interactions was restricted to myogenic cells, as forced expression of ICAM1 by fibroblasts did not augment their fusion to ICAM1-positive myoblasts/myotubes [28].

MMP9 is involved in macrophage fusion. The IL-4 induced fusion of macrophages was reduced in the presence of MMP9 function-blocking antibodies and similar effects were also observed in MMP9 null-macrophages, clearly showing the impact of MMP9 in macrophage fusion [40]. Likewise, MMP9, E-cadherin and DC-STAMP were upregulated by both IL-4 signalling and DAP12 signalling in macrophages and DAP12 overexpression induced macrophage fusion [39]. Conversely, DAP12 deficiency was associated with an impaired frequency of macrophage fusion because of low MMP9 levels [39]. Low MMP9 expression levels, concomitant with a decreased macrophage fusion rate, were also found in MCP-1/CCL2-null mice [16]. Moreover, the impaired fusion capacity of MCP-1/CCL2-null macrophages is rescued by exogenous TNF-α and TNF-α-induced MMP9 expression [16], which agrees with our data. The finding that TNF-α could induce fusion via an MMP9-dependent mechanism is further supported by data showing that the fusion of osteoclasts in bone explants, which were stimulated by human breast cancer cells through TNF-α secretion, could be blocked by the inhibition of MMP9 [41].

Even though these findings indicate a correlation between the induction of MMP9 expression and cell fusion, the detailed mechanism of how MMP9 is involved in the merging of plasma membranes still remains unclear. Cell fusion is a multi-step process that can be subdivided into i) priming, ii) chemotaxis, iii) adhesion, iv) fusion, and v) post-fusion. IL-4 and DAP12 signalling program cells into a fusion-competent status [4, 39], whereas MCP-1/CCL2 signalling is associated with chemotaxis [4, 16]. Because TNF-α induces cell fusion, it might be capable of programming cells into a fusion-competent status. Because of its proteolytic activity, MMP9 may degrade extra-cellular matrix components, promote interaction with cell membranes or may induce signal molecules necessary for cell fusion [4, 40]. We wondered whether MMP9 may degrade membrane-bound ICAM1, which may allow the plasma membranes of two cells to come into close contact. Tsai and colleagues recently demonstrated that TNF-α induced an MMP9-dependent release of soluble ICAM1 release in osteoblast-like MC3T3-E1 cells [31]. However, increased levels of soluble ICAM1 were not observed in TNF-α-treated cells nor was the TNF-α induced fusion of the cells inhibited by an ICAM1 blocking antibody.

Whether CCL2 might be involved in the TNF-α-induced fusion of M13SV1-Cre and MDA-MB-435-pFDR1 cells remains unclear. Markedly increased CCL2 expression levels were only observed in TNF-α-stimulated MDA-MB-435-pFDR1 cells. CCL2 might conceivably be involved in the induction of chemotaxis in M13SV1-Cre cells.

The finding that MMP9 plays a role in the TNF-α-induced fusion of M13SV1-Cre and MDA-MB-435-pFDR1 cells was further supported by minocycline studies, which revealed markedly lower MMP9 expression levels and a reduced fusion rate in the presence of TNF-α. This finding agrees with in vitro and in vivo studies that show that both tumour growth and osseous metastasis of breast cancer cells was effectively reduced by minocycline because of its inhibition of MMP9 expression [42–44]. It is well-known that besides its bacteriostatic efficacy against both gram-positive and gram-negative bacteria, minocycline also possesses anti-inflammatory properties (for review see: [25]). The mechanisms involved in the anti-inflammatory activity of minocycline include its inhibitory effects on the activity of enzymes such as iNOS, the MMPs or COX2, the inhibition of apoptosis and the inhibition of immune cell activation and proliferation [25]. Several lines of evidence indicated that minocycline exerted its inhibitory effects mainly through the inhibition of the NF-κB pathway [45–48]. Minocycline attenuates bone cancer pain in rats by significantly decreasing both the total and nuclear expression of NF-κB and p-IKKα in astrocytes [46]. Consistent with these findings, minocycline significantly suppresses IKKα/β phosphorylation in LPS-stimulated THP-1 monocytic cells, suggesting that it inhibits NF-κB signalling at the level of IKKα/β phosphorylation [48]. This agrees with the data that show that minocycline suppresses constitutive NF-κB activation in OVCAR-3 and SKOV-3 ovarian carcinoma cells, which is correlated with the attenuation of IKK activation [47]. Interestingly, further data revealed that the minocycline-induced suppression of NF-κB activity was mediated, in part, through the inhibition of TGF-β1 [47]. It is well-known that TNF-α signals via NF-κB [49]. Hence, minocycline most likely impairs TNF-α-induced cell fusion by blocking MMP9 expression in M13SV1-Cre cells because of the inhibition of NF-κB signalling.

## Conclusions

Our data indicate that the matrix metalloproteinase MMP9 is involved in the TNF-α-induced fusion of M13SV1-Cre cells and MDA-MB-435-pFDR1 cells. Likewise, the tetracycline-based antibiotic minocycline effectively impaired the TNF-α-induced fusion of the cells because of the inhibition of MMP9 expression.

## Additional files

Additional file 1: Differentially-regulated genes in TNF-α-treated M13SV1-Cre and MDA-MB-435-pFDR1 cells under normoxic and hypoxic conditions. Genes that were up-regulated and down-regulated by at least 2-fold in TNF-α-treated cells compared with untreated cells (not detected flags and compromised spots were not removed). Genes shown in Fig. 1 are marked in yellow. (XLSX 29 kb)

Additional file 2: Proliferation of M13SV1-Cre and MDA-MB-435-oFDR1 cells was impaired in a dose-dependent manner by minocycline. Cells were cultivated for up to 72 h with different minocycline concentrations. The data shown are the mean ± SD of three independent experiments. (TIFF 478 kb)

## Abbreviations

ABCC6: ATP binding cassette subfamily C member 6
ADAMTS9: A disintegrin and metalloproteinase with thrombospondin motifs 9
BMDCs: Bone marrow-derived stem cells
CCL2: CC-chemokine ligand 2
CLDN1: Claudin-1
COL27A1: Collagen type XXVII alpha 1 chain
DC-STAMP: Dendritic cell-specific transmembrane antigen
DMEM: Dulbecco’s Minimal Essential Medium
EGF: Epidermal growth factor
EMA: Epithelial membrane antigen
ESA: Epithelial specific antigen
FCS: Foetal calf serum
FDR: Fluorescence double reporter
ICAM1: Intercellular cell adhesion molecule 1
ICAM2: Intercellular cell adhesion molecule 2
ICAM4: Intercellular cell adhesion molecule 4
IL-13: Interleukin-13
IL32: Interleukin 32
IL-4: Interleukin-4
IL4I1: Interleukin 4 induced 1
IL7R: Interleukin 7 receptor
LAMB3: Laminin subunit beta 3
LAMC2: Laminin subunit gamma 2
lncRNA: Long non-coding RNA
M-CSF: Macrophage-colony stimulating factor
miRNA: microRNA
MMP9: Matrix metalloproteinase 9
MUC4: Mucin 4
NF-κB: Nuclear factor-kappa B
OC-STAMP: Osteoclast stimulatory transmembrane antigen
PBS: Phosphate-buffered saline
PCR: Polymerase chain reaction
qPCR: Quantitative polymerase chain reaction
RANKL: Receptor activator of nuclear factor kappa-B ligand
RIN: RNA integrity number
SDS-PAGE: Sodium dodecyl sulphate-polyacrylamide gel electrophoresis
sICAM1: Soluble intercellular cell adhesion molecule 1
SIRPα: Signal-regulatory protein alpha
TNF-α: Tumour necrosis factor-α
TNFAIP3: Tumour necrosis factor-alpha induced protein 3
VEGFC: Vascular endothelial growth factor C
ZBAF: Centre for Biomedical Education and Research
