# Supplementary figures and images for: Matrix metalloproteinase-9 (MMP9) is involved in the TNF-α-induced fusion of human M13SV1-Cre breast epithelial cells and human MDA-MB-435-pFDR1 cancer cells
Source: Cell Commun Signal. 2018 Apr 10;16:14. doi: 10.1186/s12964-018-0226-1 (PMC5894245; doi:10.1186/s12964-018-0226-1)

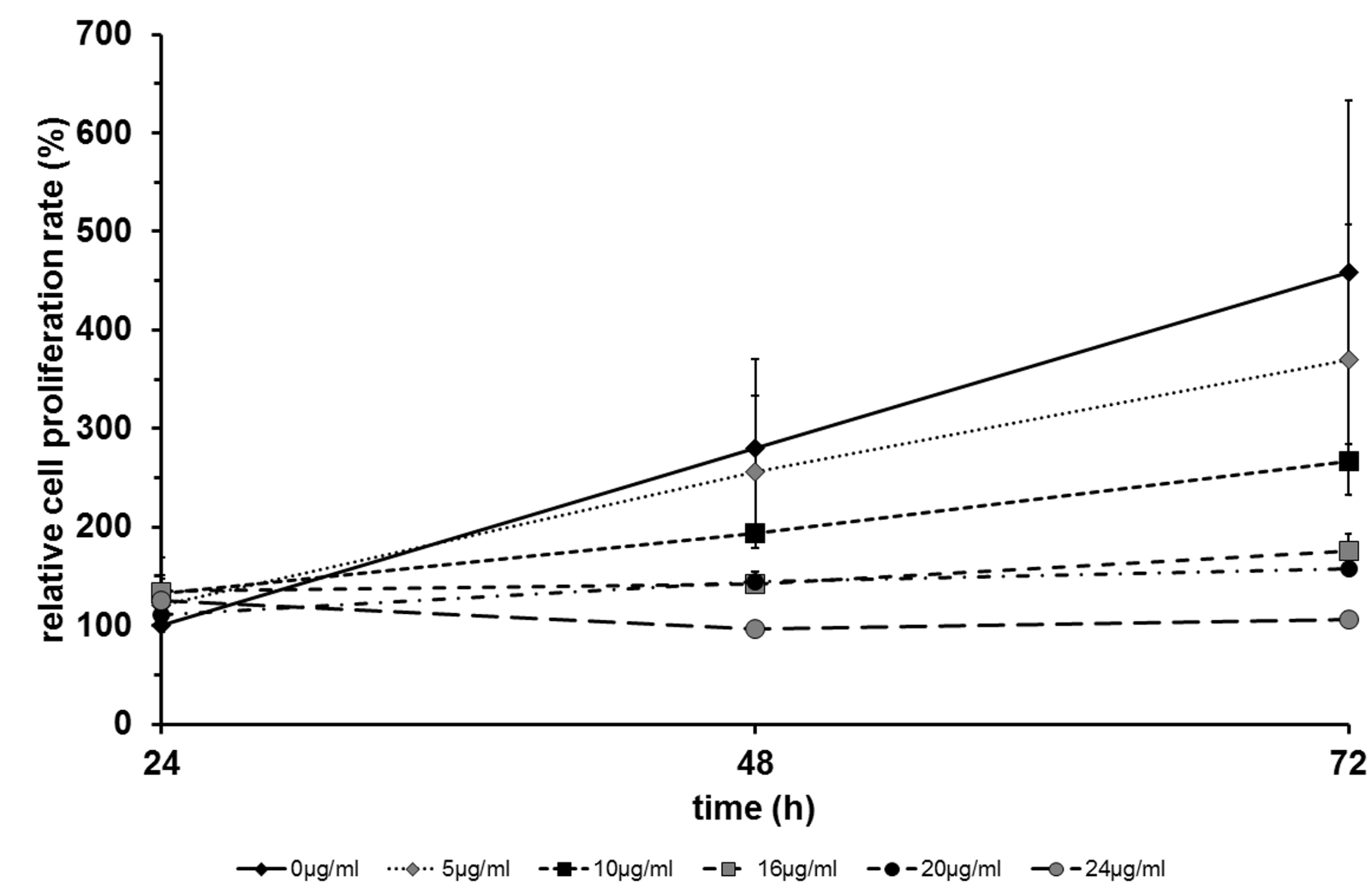

Supplement: Supplementary file 2 — Proliferation of M13SV1-Cre and MDA-MB-435-oFDR1 cells was impaired in a dose-dependent manner by minocycline. Cells were cultivated for up to 72 h with different minocycline concentrations. The data shown are the mean ± SD of three independent experiments. (TIFF 478 kb) [file 12964_2018_226_MOESM2_ESM.tif]
